# Identification of challenges and leveraging mHealth technology, with need-based solutions to empower self-management in type 2 diabetes: a qualitative study

**DOI:** 10.1186/s13098-024-01414-9

**Published:** 2024-07-30

**Authors:** Sherize Merlin Dsouza, Julien Venne, Sahana Shetty, Helmut Brand

**Affiliations:** 1 Department of Health Policy, Prasanna School of Public Health, Sherize Merlin Dsouza, Manipal Academy of Higher Education, Manipal, Karnataka India; 2 Social and Health Innovation, Prasanna School of Public Health, Manipal Academy of Higher Education, Manipal, Karnataka India; 3 Department of Endocrinology, Kasturba Medical College, Manipal Academy of Higher Education, Manipal, Karnataka India; 4 Department of International Health, Care and Public Health Research Institute – CAPHRI, Faculty of Health Medicine and Life Sciences, Maastricht University, Maastricht, The Netherlands; 5 Prasanna School of Public Health, Manipal Academy of Higher Education, Manipal, Udupi, Karnataka 576104 India

**Keywords:** Type 2 diabetes, Diabetes self-management, mHealth solutions, Digital health solutions

## Abstract

**Introduction:**

Effective diabetes management relies mainly on an individual’s ability to perform self-care tasks. However, this process is influenced by a complex interplay of factors. This study explores the multifaceted influences on Diabetes Self-Management (DSM), examining both factors influencing and affecting DSM. Understanding these influences is crucial for developing targeted Digital Health Interventions that empower individuals with diabetes to achieve successful self-management.

**Objectives:**

To identify problems faced by Type 2 Diabetes Mellitus (T2DM) individuals in self-managing diabetes and leveraging mHealth technology, with need-based solutions to Empower Self-Management in T2DM.

**Methodology:**

In-depth semi-structured interviews were conducted among ten patients with T2DM visiting the outpatient department of a tertiary care hospital in coastal Karnataka. Additionally, six healthcare professionals (HCPs) working closely with T2DM patients were interviewed to understand their perspectives on using mHealth to manage T2DM effectively. The themes for the solutions described were analyzed using ATLAS-TI software.

**Results:**

Our research examined certain factors that might have influenced effective diabetes self-management and investigated patient perspectives on using digital health solutions in diabetes self-management. This study found that technology skills, duration of diabetes, knowledge, and personal beliefs were all significant factors affecting self-management in participants with T2DM. Additionally, socioeconomic factors were also seen to influence effective diabetes self-management. The Google search engine was used by 50% of the participants interviewed to learn about T2DM. Diet management through Google searches was used by a minority (30%) of the patients. None of the participants had previously used any mobile health applications (mHealth apps) to manage T2DM. 20% of the participants expressed limited knowledge about using smartphones or wearables to track health parameters. The study also identified potential non-technological barriers to mHealth adoption. To address these concerns, researchers used an empathy map to develop solutions that promote mHealth use.

**Conclusion:**

Several challenges and need-based mHealth solutions were identified to empower diabetes self-management education among T2DM patients. Implementing need-based mHealth solutions such as data tracking, personalized feedback, and access to educational resources can lead to better disease control and a higher quality of life for those with T2DM. Further research and development in mHealth interventions, and collaborative efforts among healthcare providers, patients, and technology developers, hold a promising future for the healthcare sector in providing efficient, effective, and accessible care.

**Supplementary Information:**

The online version contains supplementary material available at 10.1186/s13098-024-01414-9.

## Introduction

### Global scenario

The number of individuals with Type 2 Diabetes Mellitus (T2DM) has been steadily increasing over the past few decades, nearly doubling since 1980, Approximately 57% of those affected remain undiagnosed [1, 2]. This rise is particularly alarming in low- and middle-income countries, where it is outpacing the increase in high-income countries. Projections estimate the prevalence to reach 7,079 cases per 100,000 individuals by 2030, reflecting a continued rise across the globe [2, 3].

Early diagnosis is important to prevent the complications associated with T2DM. Inspired by the World Health Organization’s (WHO) Global Diabetes Compact, our study seeks to understand the self-management needs of diabetic patients [4]. This aligns with the Compact’s vision of reducing diabetes risk and ensuring equitable access to quality care, while also contributing to the prevention of T2DM through improved self-management strategies.

### Indian scenario

India is the second-highest country with individuals suffering from diabetes globally, following only China. As of 2019, 77 million individuals in India are estimated to have diabetes, with T2DM accounting for the majority [5]. It’s concerning that nearly 57% of adults with diabetes in India remain undiagnosed, representing approximately 43.9 million individuals. The prevalence of diabetes in India has been steadily increasing over the past several decades and is expected to continue rising in the future. The rise in diabetes prevalence is attributed to various factors, including increasing urbanization, unhealthy dietary habits, and physical inactivity [6].

### Current scenario of mHealth apps in diabetes self-management (DSM)

The mHealth apps have become a valuable tool for DSM. These apps offer a wide range of features to help people with diabetes track their blood sugar levels, manage their medications, and make healthy lifestyle choices. Globally, several studies have proven the effectiveness of apps in the self-management of T2DM [7–10]. Participants who are given interventions achieving significantly greater reductions in HbA1c levels and weight compared to control groups. In India, and South Asian countries including India, limited studies undertaken have proven to have a significant drop in HbA1c/Fasting blood sugar (FBS) levels post-intervention [11–13].

Few of the systematic reviews and trials conducted have proven the effects of a scientific mHealth app, although the effectiveness of mHealth interventions varied widely among some of the studies [14, 15]. Some of the reviews [16], concluded that mHealth was a feasible option and had the potential to improve patient health when compared with standard care, especially for glycemic control.

### Research gaps

Current research on the mHealth app’s use for effective DSM among T2DM individuals in India remains limited. A retrospective, case-control study was conducted to assess the improvement in glycemic control in patients with type 2 diabetes using an interactive mHealth app in a population aged between 45 and 60 years in India [12]. Another study conducted, investigated the real-world effectiveness of the digital therapeutic mHealth app for improving glycemic control among the South Asian population where the data were analyzed retrospectively [13].

Hence, further research encompassing a wider range of demographics and methodologies is crucial to fully understand the impact of mHealth apps for T2DM self-management among Indian populations. It has also been reported in a systematic review [17], that diabetes management interventions targeted among South Asian patients are heterogeneous, yielding variable and limited success in reducing HbA_1_c levels, as India is a country with diversity in geographical, sociocultural, and infrastructural attributes, every intervention yield a difference of opinion among the researchers. Therefore, a need for a qualitative study focusing on patient and health care personnel (HCPs) perspectives to understand the barriers and the utility of mHealth applications among Indian diabetic patients.

### Common challenges in using mHealth as a DSM approach among patients

Ensuring access to mHealth solutions for all individuals regardless of socioeconomic factors remains a critical challenge.

While digital health solutions can aid in DSM among T2DM, Individual factors can hinder their effectiveness. People with limited digital literacy may struggle with apps and navigating functions. A lack of trust and transparency can make users hesitant [18], further, language barriers limit accessibility for users and limit their potential reach. Most importantly, even with the right tools, long-term success requires users to actively participate in behavior changes and stay motivated to maintain healthy habits for optimal health [19].

Various factors like work schedules, social norms, and personal preferences can be some of the obstacles to achieving effective DSM [20, 21]. Certain other components like the patient’s education level, age, etc., also can impact the ability to understand and implement DSM strategies. Unreliable online information can be a concern in the pathway to achieving effective DSM and hindering progress [22, 23].

Another important factor for effective DSM is Diabetic Health Literacy (DHL), DHL refers to an individual’s ability to obtain, process, and understand information related to their diabetes diagnosis, treatment plan, and self-management strategies [24].

DHL while incorporated via mHealth apps might yield better outcomes in managing diabetes-related health outcomes, actively enhancing patients’ ability to judge and decide their treatment actions and options on their self-management abilities aiding in early prevention and disease reversal, promotion of health, and prevention of complications [25].

Effective self-management practices, fostered by better health literacy, help to prevent or delay diabetes-related complications such as heart disease, blindness, and kidney failure [26].

### Future scope of mHealth adoption in DSM

The mHealth interventions may be a valuable strategy to improve diabetes care and reduce associated costs, and health impacts. The need to emphasize the adoption of mHealth applications to self-manage diabetes is now. While healthcare professional recommendations could increase app usage further, a clear trend towards mHealth acceptance is evident. This suggests that mHealth interventions could play a significant role in improving DSM and decreasing associated health complications [27].

### Purpose of the study

- The purpose of this study was to identify challenges to Diabetes Self-Management among patients by carrying out in-depth- semi-structured interviews among T2DM patients and their healthcare providers.
- Leveraging mHealth technology, with need-based solutions to Empower Self-Management in T2DM.

## Methodology

### Inclusion criteria for T2DM patients

1. People with T2DM aged 18 years and above. Gender- both males and females, Literate- able to read and write and use smartphones daily.
2. Clinical criteria for inclusion given as per the World Health Organization.

- “HbA1c ≥ 6.5%.
- Fasting Plasma Glucose ≥ 126 mg/dl.
- 2-h postprandial glucose ≥ 200 mg/dl.
- Any time postprandial glucose ≥ 200 mg/dl”.

### Exclusion criteria

1. Individuals who do not own a smartphone.
2. Individuals who are unable to read (English and Kannada).
3. Individuals with severe mental health disorders.
4. Individuals with Critical illness – “A disease or state in which death is possible or imminent” like end-stage of any cardiac, neurotic, or renal diseases including cancer and AIDS (US National Library of Medicine).

### Inclusion criteria for HCPs

1. Doctors - who completed a basic MBBS degree and who treat diabetic patients.
2. Nurses- who completed a B.Sc. degree in Nursing and are working closely with T2DM patients.

### Study design & sampling approach

In-depth, semi-structured, face-to-face interviews were conducted in person among ten T2DM patients and six HCPs using a purposive sampling approach. Six HCPs including 4 treating physicians and 2 nurses were interviewed to get their perspectives on using a digital health solution among T2DM patients.

### Ethical approval

Institutional Ethical Committee approval was obtained before the study was initiated. Participant Information Sheet (PIS) and Informed Consent (IC) were provided to the participants during the recruitment process and before the audio recording of the interviews.

### Development and validation of the interview guide

Two interview guides were developed by the researcher, one for patients and one for HCPs. The developed interview guide was validated by two subject expert teams and two professors.

Description of the patient Interview guide:

The T2DM patient interview guide consisted of two domains. In the first part, participant characteristics (Refer: Table 3), including demographic data, like patient name, age, gender, education, residence, occupation, and income were assessed. The second part covered topics to assess barriers to self-management of T2DM individuals, the use of digital health technology in assisting patients in daily activities, and the patient’s perspectives on the use of mHealth apps (Refer: Table 1).

**Table 1:** Patient interview guide

Sl. No.	Interview guide to T2DM patients
1.	How did you know about your condition? Was it an accidental or intentional diagnosis? What practitioner did you meet?
2.	How do you get in touch with your doctor for diabetes treatment? Where did you access the information about the practitioner as well as on Diabetes? Name sites if any? From whom have you heard?
3.	How do you manage diabetes in your day-to-day life?
4.	Do you use any diabetic apps/ has anyone in your house using a diabetic app? If yes for how long?
5.	What is your opinion on using a mHealth app for managing Diabetes?
6.	What are your concerns about using a digital platform for feeding your health details?
7.	Do you face any challenges in managing your diabetes? if yes, what are those?
8.	What kind and how did you receive information about your disease and its management? Was that helpful? Or do you feel some information was missing? Which ones?
9.	Do you feel confident, or do you experience stress when managing your diabetes?
10.	Do you record the daily measures you’re doing? Is it easy?

### Description of the HCPs interview guide

The interview guide for the HCPs also consisted of 2 domains, the participant characteristics, like the HCP’s name, education, and affiliation (Refer: Table 2). The second part included open-ended questions addressing the DSM pattern followed among the T2DM, the patient’s use of digital health technology in self-managing diabetes, and the HCP’s perspectives on utilizing mHealth apps among the patients (Refer: Table 3).

**Table 2:** HCPs Profile

Sl. No	Qualification	Work affiliation
1.	MBBS	Doctor
2.	MBBS	Doctor
3.	MBBS	Doctor
4.	MBBS	Doctor
5.	BSN	Nurse
6.	BSN	Nurse

**Table 3:** HCPs interview guide

Sl. No.	Interview guide to the HCPs on Type 2 diabetes management among patients
1.	Could you describe the patient’s journey from their 1st visit/diagnostic (frequency of visits, other professionals intervening)
2.	What are the most frequently asked questions? Most frequent concerns?
3.	Which resources do they use more often? What kind of help do they find around them?
4.	What percentage of patients do you think are unable to access information/ get treated during their hospital visit?
5.	What are your views about patients using the mHealth app for diabetes management?
6.	What are your views on using a diabetic self-management app?
7.	What do you think are the advantages and disadvantages of using an m app for the management of type 2 diabetes?

#### Interview setting

Interviews were conducted at the Outpatient unit of the Endocrinology Department of a tertiary care hospital in Karnataka, India. The researcher obtained written consent from the patients and the HCPs to be a voluntary part of the study. The consent form was also given in the local language to the patients for their understanding of the study criteria and inclusion. The study purpose was explained to the HCPs, and the patients, and consent to audio-record the interview was also taken.

#### Interview procedure

Face-to-face, one-on-one Interviews were conducted after obtaining patient consent to conduct the study on them. Interviews were conducted in a private room of the hospital in the presence of a diabetes counselor who was also the gatekeeper for this study. The researcher recruited participants fulfilling the study inclusion criteria. When necessary, field notes were taken to note information on the patients and the HCPs and to ensure that the participant’s basic information was not missed.

Similarly, the HCPs included in the interviews were the doctors(*n* = 4) and the nurses (*n* = 2). Here, the doctor in charge was the gatekeeper in introducing the participants to the researcher who fulfilled the study inclusion criteria. The interviews among the doctors were conducted one-on-one in the doctor’s consultation room with their permission after obtaining their consent to audio-record the interviews. The audio-recorded interviews among the nurses were conducted in the nurse’s education room after obtaining their consent in the presence of a diabetes counselor.

The audio recording was conducted by the interviewer/researcher using a phone. The demographic details were asked of the patients and the HCPs. The average duration of each interview conducted among the T2DM patients and the HCPs lasted 30–40 min. To ensure a comprehensive understanding, interviews continued until no significant new themes emerged. Similarly, participant recruitment stopped upon reaching data saturation, signifying a sufficient sample size to capture the full range of experiences among both patients and the HCPs.

### Data transcription and translation and analysis

Transcripts from the recordings combined with field notes from the researcher were used in an iterative process. The interviews were conducted in the local language Kannada, among the T2DM patients for their ease and understanding, the medium of language English was used for the interviews conducted among the HCPs. To ensure an accurate capture of participant experiences. The bilingual researcher conducted a verbatim translation of the audio recordings from Kannada to English. The translated interviews were transcribed by the researcher and coded using the Software ATLAS TI.

The researcher finalized the coded themes with the other research team members to ensure their accuracy. The coded data was then compiled into final primary themes. The patterns found in the qualitative interview data were used to identify and group descriptive codes.

For the final codebook, paraphrases from the transcripts were described for each theme and subcategories.

Deductive thematic analysis is used to identify common themes and subthemes from the interviews conducted among individuals suffering from T2DM. Later their views were systematically compared on the factors affecting optimal DSM. Perceptions of the HCPs on the use of Digital health solutions for effective DSM among T2DM individuals were narratively summarized. The common themes from the patient interviews were grouped and reported. The mHealth solutions with appropriate features adaptable to the patient’s problems/ needs in the current situation/settings were identified and addressed by the researcher simultaneously.

### Framework

We have adopted the first two phases of the “Design Thinking Model (DTM)”, which are to empathize and define the problem statement, that gives a focus on the user needs that we are trying to identify through our interviews.

The first 2 phases of the DTM will be detailed to empathize and define the problems faced by T2DM individuals in managing diabetes.

1. **To Empathize**: The user needs (T2DM patients) were identified through the interviews.

*Using an Empathy Map to describe the process*:

Empathy Map is a tool that helps us to empathize and synthesize the observations from the research phase and draw out unexpected insights about usersneeds. Empathy Map allows us to sum up the learning from engagements with people in the field of design research.

2. **To Define**: State User’s needs and problems.

The data obtained from the interviews at the initial stage were transcribed and the user stories were used to depict and conclude a specific feature that will be used in the ideate phase. User stories are a few sentences in simple language that outline the desired outcome. They don’t go into detail. Requirements are added later, once agreed upon by the team [28].

User stories are structured as personas as detailed below.

As a persona: The researcher is trying to understand who the person is and understand how that person works, how they think, and what they feel. And has the feeling of empathy for that person.

Wants to: here we try to understand what they need or what they are trying to achieve. By describing their intent and not the features that they use.

So that: what benefit, or outcome are they trying to achieve, and what is the problem that needs to be solved to achieve what they want?

We will use an empathy map to explain the above-mentioned 2 phases of DTM, where the user stories will be mapped to get insight, that enables us to start designing potential solutions. The persona discussed in detail here is the patients- what difficulties they face while trying to manage their condition and giving out the best possible solutions according to their needs.

## Results

### Demographic characteristics of the patients

(Refer: Table 4- Demographic details of T2DM patients)

- Age: most of the interviewees belonged to the age group 35–60, with a mean age of 48 years.
- Education: The participant’s educational qualification ranges from SSLC to a bachelor’s degree. T2DM individuals with better educational qualifications bachelor’s degree *n* = 3/10.
- Gender: The study found that managing diabetes was particularly difficult for male participants who were the primary income earners for their families (80% of participants). This suggests a potential conflict between work demands and healthy self-management practices for male breadwinners.
- Occupation: The study showed that retired participants consistently followed DSM routines. This suggests that having a more flexible schedule might be beneficial for managing diabetes.
- Family income: The study discovered that among participants who belonged to the low-income group (earning less than 3 lakhs annually/ $3593.54/year), the cost was a significant barrier to effective DSM. One participant specifically mentioned rationing glucose strips due to their perceived expense.
- Duration of diabetes: The study suggests a link between consistent self-management and long-term glycemic control. Two participants with chronic diabetes who regularly monitored their blood sugar levels showed good adherence to self-management routines. Additionally, another participant diagnosed for over two years also demonstrated consistent self-management. All the participants recruited in this study belonged to rural areas of Karnataka state.

**Table 4:** Demographic details of T2DM patients

Sl. No.	Age in years	Gender	Education	Occupation	Annual family income in lakhs	Duration of diabetes in years
1.	59	Male	SSLC	Farmer	< 1.5	> 1
2.	66	Male,	PUC	Retired hotel worker	< 1	> 10
3.	78	Male	Graduate	Retired bank employee	< 1	> 10
4.	32	Male	Graduate	Business	< 2	< 1
5.	35	Male	Graduate	Postmaster	> 2.8	< 1
6.	38	Male	Graduate	Private taxi driver	< 2	< 1
7.	40	Male	PUC	Bus conductor	< 2.4	< 1
8.	50	Female	SSLC	Homemaker	< 1.3	< 1
9.	44	Female	PUC	Homemaker	< 1.8	> 2
10.	38	Female	SSLC	Homemaker	< 3	> 1

**Fig. 1:**
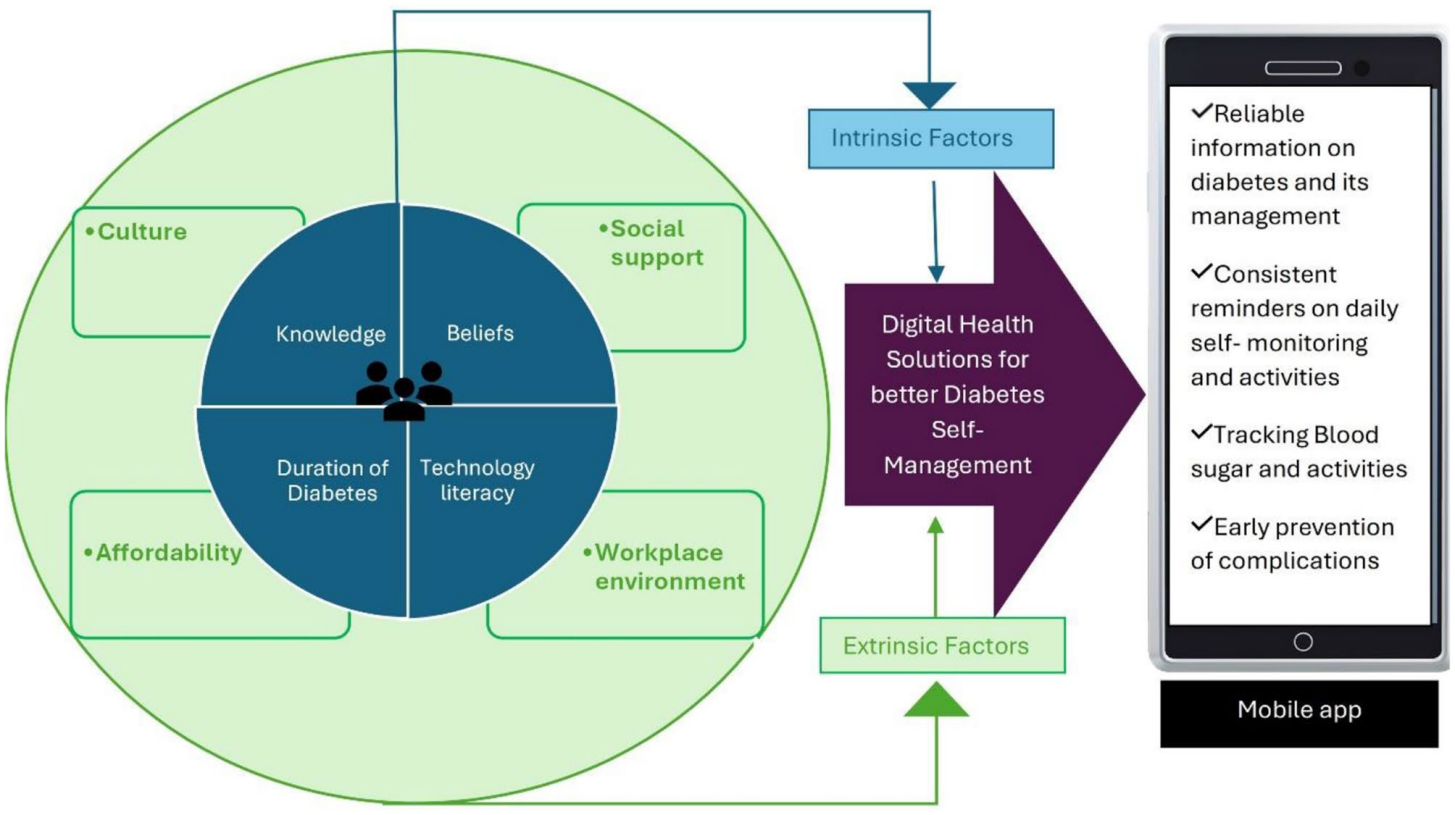
Empathy map showing the factors influencing effective DSM among T2DM individuals and strategies to simplify their needs

We have detailed perspectives of T2DM patients on the factors associated with self-managing diabetes, as well as understanding their needs using an ‘Empathy map’ given below in (Refer: Fig. 1).

### Extrinsic factors influencing diabetes management among T2DM individuals: elaborating Fig. 1 below

*[P – Indicates Patient responses]*

#### Socioeconomic factors

- **Culture**: South Indian culture presents a unique challenge for T2DM patients due to the staple foods such as rice, rich in carbohydrates. Cultural preference for food among the south Indians clashes with traditional diabetic dietary advice given by their providers, making it difficult for individuals to manage their blood sugar through diet alone. Interviews revealed participant’s struggles in avoiding rice, highlighting the need for culturally sensitive approaches to T2DM management in South India.

*P9- I feel I don’t have clarity on exactly the diet*, * I went to the dietician she has given me a chart to follow the diet. But still*, * I have carb items for my breakfast and diet.*

To cater to the cultural needs, there is a need for culturally sensitive mHealth apps that provide healthy alternatives to traditional food ensuring low carbohydrate content, without compromising DSM.

b. **Social Support**: The study identified social support as a crucial factor in T2DM management. Family members had a significant role in helping patients to navigate online resources, for example using a Google search engine to understand diabetes and find information on managing nutrition. While 50% (*n* = 5) appreciated support from doctors and HCPs, 40% (*n* = 4) highlighted the positive impact of family and peers. The family support system assisted with medication adherence, knowledge acquisition, and overall, self-management. However, despite strong support from physicians, family, and friends, challenges persisted in managing aspects like diet and exercise.

*Quote on supportive family: P8- I don’t know how to use mobile phones; my children help me with it.*

The study highlighted the importance of social support from family members in encouraging the use of phones, similar motivators from close family circles and HCPs could build trust and relieve anxiety among the individuals. By fostering motivation information sharing and accountability, social support can empower individuals to manage diabetes through mHealth apps,

c. **Workplace environment**: Time constraints emerged as a significant barrier, particularly for the breadwinner of the family. Busy work schedules often faced by male participants, hindered effective diabetes management. Similarly, some female participants, primarily responsible for household chores have also reported challenges due to time limitations.

*P7- I have no time left after my hectic work schedule.*

P5- I find it difficult to manage because I go to work and in between, I can’t concentrate to check sugars and have medicines.

P7- No, also I don’t get enough time because of my job and hence I cannot do it.

These findings highlight the need for flexible DSM strategies that include using mHealth apps to access and initiate better management that can accommodate busy lifestyles.

d. **Affordability**: The participants felt the cost of the glucometer and strips was high and as a result, they could not monitor blood glucose regularly.

Quote supporting the statement given by the study participants-

*P2- I don’t check my sugar daily; I get my legs swollen*,* then I realize my sugar has spiked and do the sugar check. Or it’s too expensive to check every time the sugar levels.*

*P2- I monitor usually but sometimes I can’t correctly see*,* so I need to use a lot of time and spend money on extra strips and needles. So*,* I don’t check often.*

Diabetes management requires various expenses including medication, blood glucose monitoring supplies, consultation fees, and quality food choices resulting in a significant financial burden on the patient or their family with limited income or inadequate health insurance facilities. The digital health solution here can be optimizing a mHealth app, with collective features including doctor appointments at a low cost, providing options for budget-friendly quality food choices, also having reliable educational content on testing blood glucose, unnecessary pricking of fingers to test, and unnecessary wasting of the strips, thus reducing the expenses incurred.

### Intrinsic factors influencing DSM among T2DM individuals

#### Knowledge of effective DSM

Knowledge of DSM is associated with daily activities such as taking medication, diet, exercise, and blood glucose monitoring. Poor knowledge of managing diabetes was found among the T2DM individual’s reasons for this were that they were unaware of their condition at the initial phase of diagnosis and how they could manage it in their daily lives. A concern reported by the T2DM individuals on difficulties faced in managing diabetes is-.

#### Confusion to manage hypo and hyperglycemic levels

Fluctuations in the glycemic levels pre- and post-meal among the individuals confused medication adjustments.

*P4- Sometimes the GRBS count comes up to 400 or 500 so then it’s a problem on what to do and how to control it. So*,* I take medications but when I take insulin then I get weak. My limbs shiver. So*,* then the doctor advised me not to continue insulin.*

This suggests a difficulty in decision-making among the patients. The need for reliable education on what to do and being prepared is crucial in this situation. The Health app could provide an educational module on fluctuations in blood glucose and how to interpret them in the context, which can be beneficial. This can empower patients to better decision-making and avoid confusion.

#### Beliefs

One of the T2DM individuals exhibits distrust of conventional medicine. The patient was likely to be concerned over the potential side effects of medications and particularly believed that they would eventually get their kidneys damaged. They would rather try Ayurvedic treatment for their cure (a traditional Indian system of medicine known for its focus on natural remedies and holistic well-being).

***P 2- ****I first began with the ayurvedic med and then I shifted to allopathic.*

Educating patients on the limitations of other kinds of treatment from a healthcare provider is necessary and communicating this problem with a larger population addressing patient’s concerns and explaining benefits using digital platforms like mHealth apps or media could be effective in reaching the desired population in aiding them to make an informed decision.

#### Technology literacy

A. Poor technology literacy: Particularly elderly individuals who may not have grown up with modern technology or had limited exposure to it. The study revealed a digital divide among the older adults with T2DM. Limited technology literacy made it difficult for them to navigate search or utilize search engines effectively. This made them dependent on the younger generation, family, and friends to access the information and hindered their ability to access and manage diabetes independently and effectively. Eventually, as reported by some of the participants, after a dependent phase on family members to access diabetes-related information by navigating online and making their self-decisions on certain food choices.

*P1- I don’t know how to use it; I must take my kids’ help for it. I check my GRBS daily so I must control my diabetes and I will ask my Daughter-in-law’s help for it in the future if I use it (non-app user).*

Health Information Seeking Behaviour (HISB) refers to How individuals seek information about their health, risks, illnesses, and health-protective behaviors (Lambert & Loiselle, 2007; Mills & Todorova, 2016) [29, 30].

Health-related information seekers and chronic diabetics were better at testing their blood glucose regularly and managing diabetes. Surprisingly, individuals irrespective of age, gender, and marital status, with higher income, were associated with online health-related information-seeking behaviors.

*P4- Yes*,* I searched Google and looked for what diet to eat*,* and hence I have adopted a vegetarian diet. And my brother taught me about doing an online search a year back. Now I google search myself.*

T2DM individuals preferred using online platforms like Google search engine to get a deeper insight into the disease and its management. The use of Google search was helpful for 4 participants mainly in planning and managing their diet, practicing mild exercises like walking, and getting prepared to control diabetes complications. Learning and understanding diabetes, not only through online platforms but also through personal experiences from friend groups and newspapers was found to be effective among one of the participants.

Also, a few read articles in the newspaper on the health and wellness sector and found educational TV shows on regional TV channels hosted by physicians useful, to gain an understanding and knowledge of their disease and its management.

#### Duration of diabetes

The longer the diabetes duration, the more T2DM individuals were confident enough to self-manage diabetes via regular diet, exercise, and blood glucose monitoring as reported by three chronic patients compared to newly diagnosed young T2DM individuals.

*P9- I eat more of a veg diet*,* try to drink sugar-free tea*,* and walk for half an hour a day.*

*P3- Now*,* I am monitoring Glucose four times a day and recording. Now it’s under control with one week of medication they had given me at the hospital.*

The study suggests a potential association between diabetes duration and confidence in self-management. The increased confidence seen by three of the participants suggests greater openness to mHealth adoption especially in tracking blood glucose levels, medication adherence, and improved dietary choices. The inclination towards using an app can also suggest enhanced communication with doctors, as well as helping doctors make better treatment decisions.

### Digital Health Solutions addressed by the researcher according to the patient needs identified (refer: Fig. 2)

**Fig. 2:**
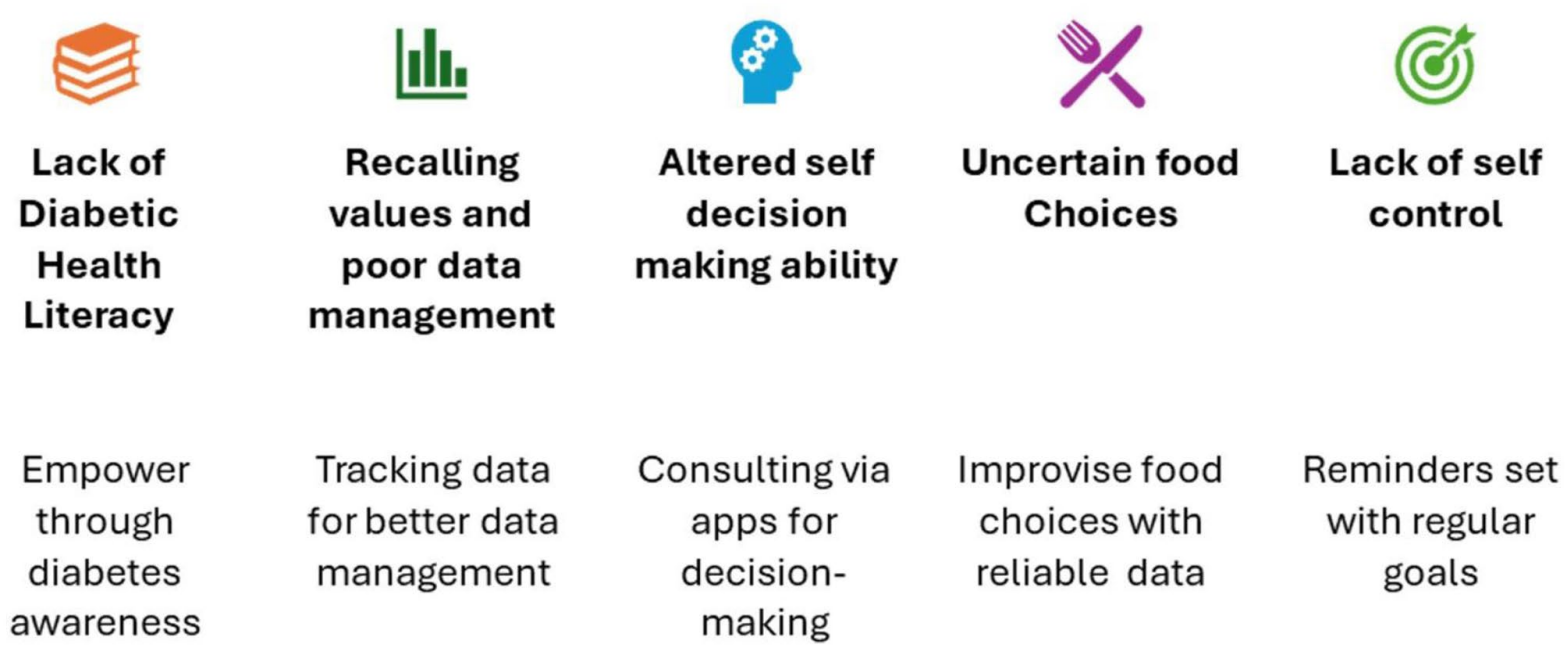
mHealth-led solutions addressed based on the patient needs identified

### Lack of diabetic health literacy

Awareness about one’s health condition, in our case scenario, T2DM is a vital aspect of initiating the actual treatment for improving health outcomes. In this study, the newly diagnosed T2DM Patients were unaware of diabetes, its causes, and symptoms, and ignored the signs of giddiness, fainting, shivering, and feeling thirsty often, unless they got diagnosed in the hospital.

The interviews conducted among patients with T2DM, who were aged 60 and above/chronic diabetics, and especially those who retired from their jobs monitored their blood sugars promptly. They gathered all the information on diabetes for years from their family, and friends and developed a good rapport with their healthcare providers. Whereas the rest of the newly diagnosed T2DM individuals were trying to figure out what was the reason for them to develop diabetes. On being newly diagnosed patients, being asked how they manage diabetes on their own? The participants were found to have problems recalling the health education they were given on managing diabetes by their providers on their first visit.

*P3- At the hospital*,* they told me- it’s late to treat! What can I do? The problem was that in the mornings my blood sugar was low but in the evening my blood sugar levels were high.*

#### Solutions

One way to address the issue of people with poor diabetic health literacy is to launch a public health initiative, such as creating and distributing diabetes education materials. Another technology-driven way is to provide easy-to-access educational materials via Digital Health apps is helpful in this technological age and provide information on the causes and risk factors of diabetes, healthy eating, weight control, blood sugar monitoring and control, medication administration, etc. Diabetes-related health education via an app can be easily accessible anytime and anywhere. This also can help reduce recall issues from the patient’s first doctor visit and recollect the information on diabetes and management leading to enhanced and elevated DSM levels.

### Problems with recalling and poor data management

The interviews we conducted among the T2DM patients highlighted poor data management of their health-related data, especially regular blood glucose monitoring, insulin dosage, or medication intake. Two of the chronic diabetic patients interviewed stated that they kept a record of their blood glucose regularly and used reminders for their next visits. But whereas the newly diagnosed diabetic patients were clueless about how to go about tracking and monitoring their health condition.

*P2- I keep track of blood sugars once every month by writing on a paper.*

*P9- I check blood sugar weekly 4 times. I have a blood sugar monitor at home and write it down in the paper that they have given me from the hospital.*

#### Solutions

The mHealth interventions like mobile applications in this situation can help track daily activities and record them to prevent forgetfulness related to age and workload and fear of the paper getting lost.

Incorporating features in the app like reminders for tracking patient medications, blood glucose levels, information on controlling diet, regular exercise, and follow-up hospital visits will be a favorable option to self-manage one’s diabetes condition. Similarly, some of the supporting articles explain a few of the benefits [31, 32].

Diabetes management also involves tracking various data points such as the patient’s daily activities like exercise or weight could be effective in determining the prognosis of the disease [33]. Few patients were also expressive about using their phones for keeping reminders and managing diabetes in their daily lives [34].

### Altered self-decision-making ability to manage diabetes

Avoiding confusion on what to do? when the blood glucose readings peak is often a concern among the interviewees as reported by them. They have reported to be trembling and confused about what to decide on their next blood glucose reading.

The need was to know the right dose of medications and methods to follow when their sugar levels peaked or dropped.

*P3- As per doc orders I take medicine regularly. But confused*

*P2- I monitor usually but sometimes I can’t correctly see*,* so I need to use a lot of time and spend money on extra strips and needles. So*,* I don’t check often.*

#### Solutions

Blood glucose levels typically peak 1–2 h after eating, depending on various factors like food composition and individual metabolism. Making the patients knowledgeable about their normal blood glucose ranges and preparing them for any emergency is crucial. Hence sharing this information using an app with updated and evident information will enable them to prevent their situation from worsening. Nevertheless, consulting their doctor or healthcare provider on call for personalized guidance is much more beneficial, hence contact information of the treating physician for crucial decision-making at this point will be helpful.

This can keep the patients prepared by knowing their target range, having readily available snacks or medications in hand, and understanding what actions to take based on their reading.

### Uncertain food choices

T2DM patients expressing their doubts and lack of clarity about what to eat was another important concern to manage diabetes effectively. Difficulty understanding dietary recommendations can lead to frustration and confusion.

For example, one of the participants in the interview was confused about the diabetic diet and mentioned that she was starving most often because she did not know what to eat. Getting diagnosed with a chronic condition like T2DM can be traumatic and stressful at the same time. Dietary restrictions can add to the burden. Patients might feel overwhelmed, frustrated, or unsure about making healthy choices.

*P8- I can’t go without having food*,* because the doctor advised me to be on a strict diet and eat limited*,* so I am fasting for a week. I only ate ragi and jowar.*

#### Solutions

The abundance of information available online and from various sources, is often confusing. Patients might struggle to distinguish reliable sources from misinformation. Medical and nutritional terms can be confusing for individuals without a scientific background. Hence including reliable evident information on healthy foods and how to prepare basic recipes is important. Using simple language, visuals, and recipe techniques is necessary with the help of mHealth apps while disseminating health education on diabetes. Active involvement of the HCPs to develop individual meal plans based on their specific needs and preferences is another non-technological solution that could be made technology-dependent with the dietician’s active involvement.

### Lack of self-control

Another problem faced by the patients is, to abide by strict monitoring and glucose control due to irregularity in daily activities performed, along with the patient’s inability to maintain a healthy diet for longer. For example, one of the participants stated that they could not resist eating food items that were not healthy and ended up eating higher quantities at a time.

### Solutions

Use an activity tracking feature in the mHealth app to track patients’ activity levels, identify patterns, and features to set reminders to continue and motivate the individuals to monitor and self-manage diabetes regularly. This can help adjust patients’ exercise routine or insulin intake based on their activity level on any given day.

The solutions for the problems faced by the patients listed above could be put forth by customizing a mHealth app with all features put together. Accurate and reliable data on diabetes conditions from various standardized national and international organizations like the American Diabetes Association (ADA), International Diabetes Federation (IDF), and the World Health Organization (WHO) at the International levels and national levels Indian Council of Medical Research (ICMR), will work towards the better health of an individual and getting optimum health outcomes. Educating individuals on the core of DSM, avoiding future complications, and early prevention for those individuals whose HbA1c values are just at border levels might help mitigate the future disease burden in India.

## Patient and provider perspectives on the use of mHealth apps/Digital health tools for diabetes self-management

### Patient perspectives on the use of the mHealth app for DSM

One of the 10 participants was found to have used a mHealth app for managing their weight, following are the supporting quotes from the patients (Refer: Table 5).

*P10- I think it can be very useful. I think the customized diet aspect will be a good option in the app to have.*

*P2- I use the app and check articles on diabetes*,* but I don’t completely follow it*,* but if in general*,* they tell me that fennel seeds are useful then I drink fennel water but don’t take all of what’s given in the article seriously. And take advice only that I feel like I must take up.*

**Table 5:** Patient and provider perspectives on app usefulness

Perspectives on the use of the mHealth App	
Patient Perspectives	Diet customization Enriching knowledge via Diabetes-related articles Aid in weight loss
Providers (HCPs) Perspectives	Regular patient monitoring Ease (educating on diabetes) Reaching a larger population at the same time. Early diagnosis of diabetes Diabetes prevention among prediabetics Preventing future diabetic complications Better time management Tracking blood glucose levels Building exercise routines among patients Avoiding diet-related confusion

Supporting quotes from the patients using digital platforms for searches

*P4: Sometimes useful but not every time*,* I used to search and buy vegetables.*

*Earlier I used to binge eat but now I control my diet.*

## Healthcare Provider’s perspectives on the use of the mHealth app for diabetes self-management among T2DM patients (refer: Fig. 3)

**Fig. 3:**
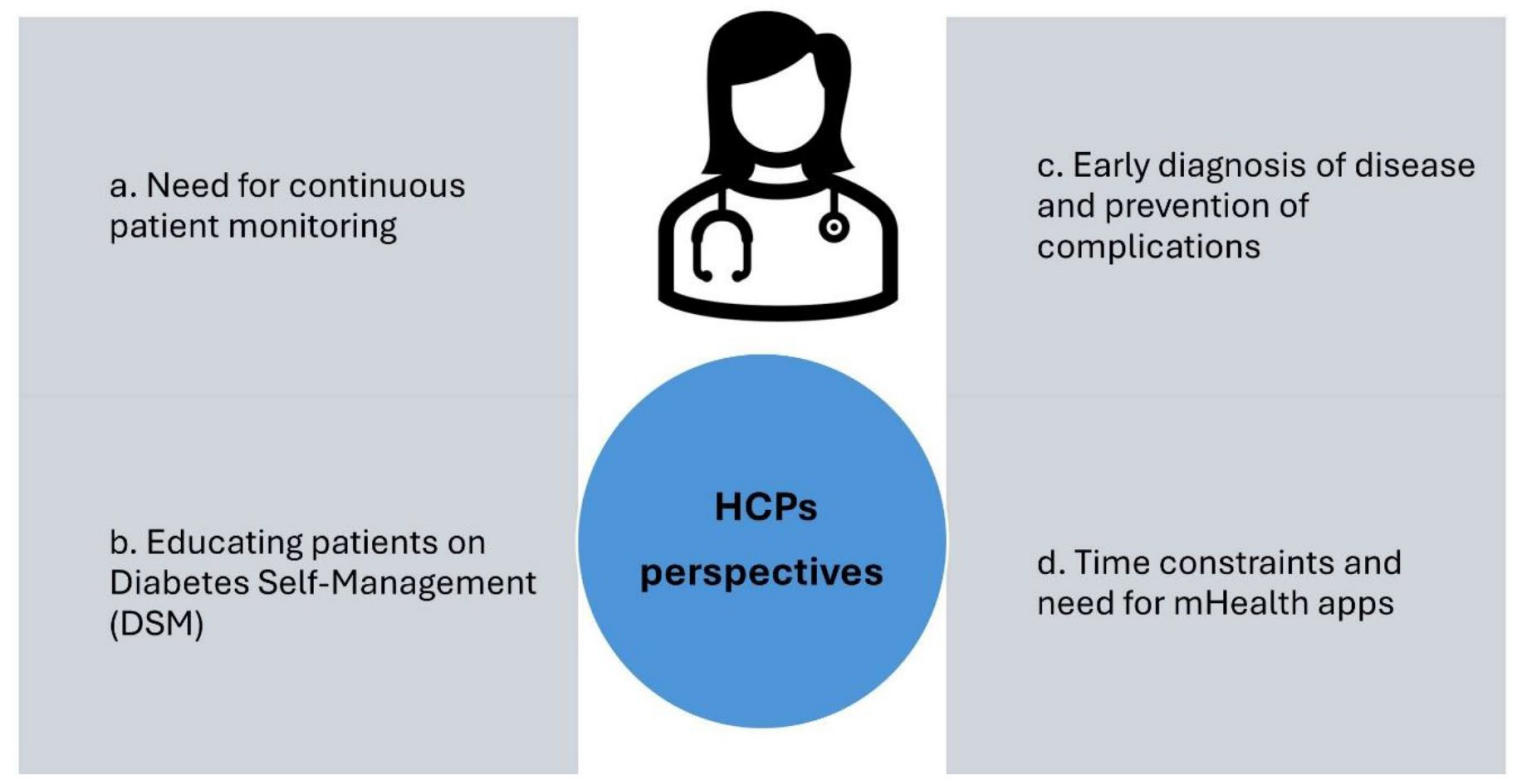
Perspectives of the HCPs

### Need for continuous patient monitoring

The professionals expressed their need for continuous monitoring of the patients; this was possible with regular efforts put forth by the healthcare team and the patients themselves (Refer: Table 5). The monitoring of blood glucose and proper insulin dosage was a crucial part of diabetes management, hence the doctors were dependent solely on the patients reporting their blood glucose levels accurately and in determining their next doses.

*[D – Indicates Doctor responses*,* N – Indicates Nurses responses]*

*D1- Most patients*,* like 60% come from far away like 2–3 h distance*,* for such people*,* we tell them to monitor GRBS at home. We give them duty mobile number if they have doubts*,* they can call us in OPD hours. From our side*,* we make sure that they have access to information*,* but I am not sure because of their illiteracy people cannot.*

*D3- One thing I have noticed is even though we explain everything on disease management the patients do not follow what we have said*,* and they are not taking anything that we have prescribed*,* and they will start with Ayurveda and homeopathy treatment.*

*For a few months or years*,* they will start taking some alternative medicines and come back with high sugars or getting complications then they return to our treatment.*

### Educating patients on diabetes self-management (DSM)

Patients tend to get confused about managing their diabetes through proper diet and exercise, some concerns stated by the professionals were.

*D1- They ask us - why my sugars are high. I do not eat rice*,* yet they are high! so dietary concerns are more.*

The foremost aspect of identifying the condition early and avoiding future complications of diabetes is to educate on the disease condition. Educating through means of mHealth apps poses a lesser burden on the HCPs. Hence, educating and reminding the patients on what to do and what not regularly helps them to understand and adapt to good behavioural habits in managing their condition effectively.

### Early diagnosis of disease and prevention of complications

It’s crucial to reach out to the population to diagnose diabetes at an early stage since it’s curable initially through lifestyle modifications. When diabetes is left untreated the blood sugar levels spike up leading to a life-threatening condition with certain complications of the heart, neurons, eyes, skin, and kidneys or also leading to amputation of the leg, hindering the patients from doing their daily activities.

*D3- Most of the patients are asymptomatic they come with a heart or a kidney problem or some bilateral tingling sensation in the feet and incidentally they are diagnosed as diabetics. So*,* there are no specific clinical features because if the sugars are 200*,* also you’re all right*,* and if it’s 500 also then the individual is asymptomatic. Most people are coming with uncontrolled diabetes like HbA1c of 10 or 11. It means that their sugars are high for a few months*,* but they won’t know it and eventually come up with other symptoms.*

Early diagnosis of diabetes is crucial for doctors to prevent future complications among their patients by identifying the condition early, doctors can implement treatment plans that focus on lifestyle changes and potentially delay or even prevent the development of serious health problems. mHealth apps hold immense potential in this area as they can empower patients to monitor their blood sugar levels, track their diet and exercise routines, and receive educational resources, all of which contribute to early awareness of symptoms. This can be particularly beneficial for patients who might miss out on in-person education sessions given by the HCPs due to scheduling conflicts or other limitations. With mHealth apps, T2DM patients can access vital information and self-management tools at their convenience, ultimately fostering better control over their diabetes.

### Time constraints

The physician’s concern was giving limited time to each patient out of patient’s hospital visits due to patient pooling at the outpatient unit at the hospital department. Patients received a maximum of half an hour of face time with the physician in a day, although the visits were not limited to the patients, ideal visits to the patients were scheduled at 2 weeks to 4 weeks or more depending on the severity of the patient’s condition.

*N6- Time is very important for each and everybody. Here also for the hospital staff*,* the doctor is only one doctor*,* he must see the IP*,* emergency*,* and OP patients*,* and along with this*,* they are pushing this also then it’s hectic for the doctor also. Time is necessary.*

#### Need for mHealth app

From the interviews conducted among HCPs, doctors, and nurses have expressed positive views on using a mobile application for diabetes management. This suggests potential benefits for both healthcare providers and patients.

*D1- I think mobile apps can be useful for patients coming from far away.*

*N5- I think mobile apps are useful for the patient*,* especially educated people*,* and may be financially liable.*

*D2- Definitely apps are useful in tracking patients’ blood sugars*,* SMBG monitoring*,* creatinine*,* cardiac values*,* diet and it’s difficult to titrate diet in calculating the calories they take would be helpful.*

Healthcare experts especially the physicians interviewed expressed the need for a common technology that can be used among the patients to self-manage T2DM and record their blood glucose levels promptly at home. The doctors could easily monitor the patient’s glucose readings at their follow-up. The physicians also felt that the confusion among the patients on diet and exercise management could be reduced if they were educated about DSM.

Digital platforms like mHealth apps can provide access to educational resources and doctors can implement digital health talks to deliver essential audiences. This can save time during in-person consultations, allowing doctors to focus on personalised patient care. Patients can ultimately receive comprehensive information at their convenience and doctors can streamline their workload, creating an efficient healthcare system.

## Discussion

Managing diabetes requires a comprehensive and collaborative approach involving healthcare professionals (HCPs) and T2DM patients. As we have identified in our study, several socioeconomic factors like culture, social support, workplace environment, and affordability can come in the way of achieving effective DSM, there are several studies conducted that also exert similar issues that we have found in this study. Certain dietary concerns and social support were also found to be the extrinsic factors identified and discussed by an author in their research study, conducted across a similar context to India [35]. Certain individual factors like working age group or students who are enrolled in academic activities are occupied full time were shown to have deficit DSM routine while compared to others who worked at home have also shown to have an effect in engagement with health-care providers or lack personal motivation in managing diabetes [36]. As there is a lack of studies conducted in India, focusing only on the cost-effective management of diabetes, a study conducted in Qatar was found to be relational to our discussion, where the cost of DSM supplies was an obstacle to effective DSM among the participants [37].

Other intrinsic factors like limited knowledge of health, limited technology literacy, beliefs, and duration of diabetes have been shown to have affected DSM. Limited knowledge and Long-term safety-related beliefs among T2DM individuals were some of the factors identified in a systematic review conducted among South Asians, and an RCT conducted in India [38, 39]. Another systematic review conducted on web-based monitoring has shown to influence the technology literacy levels, indicating definitive future research on topic [40]. Duration of Diabetes dependent level of DSM was identified as one of the factors influencing DSM from a research study conducted in South-West Nigeria [41].

As technology advances, digital health solutions have gained prominence in providing innovative tools for better diabetes management. This discussion delves into the needs of T2DM patients in simplifying their self-management abilities using digital health as a solution, the challenges faced by HCPs in managing T2DM patients, and the potential of digital health solutions in addressing these concerns.

**a. Importance of Digital Health Literacy in offering digital health as a solution**:

Digital literacy is described as “The ability to use information and communication technologies to find, evaluate, create, and communicate information, requiring both cognitive and technical skills” (American Library Association, 2017; UNESCO (United Nations Educational Scientific and Cultural Organization, 2011) Digital health literacy, at first glance, can be regarded as the convergence of digital literacy and health literacy [42, 43].

Health Literacy including digital health literacy plays a crucial role in the effective use of digital Health Solutions, particularly among individuals with T2DM. Educating patients about their condition, self-management, and lifestyle modifications is vital. HCPs need tools that engage patients and encourage them to take an active role in their care.

Digital health technologies can offer valuable resources for self-management, monitoring, and accessing relevant health information. However, the level of health literacy can significantly impact a patient’s ability to utilize mobile health technologies effectively. A few studies identified similar barriers, where numeracy and health literacy were positively correlated with digital health literacy. In another study conducted, it was discovered that lower digital health literacy was related to less regular usage of a computer to look for diabetic drugs or treatments [44, 45], Patients with high Digital Health Literacy were 1.85 times more likely to achieve target glycemic control than patients with low Digital Health Literacy, according to a cross-sectional study conducted at the University of Gondar Comprehensive Specialized Hospital in 2019 [46].

Another study showed the relationship between Digital Health Literacy among T2DM individuals to manage diabetes effectively and found that individuals with better Digital Health Literacy are more likely to comprehend the functionalities, features, and benefits of Digital health technologies. The T2DM individuals could understand the information presented and effectively navigate various tools and applications [47].

To promote the effective use of mHealth technology among T2DM patients, it is important to consider and address Digital Health Literacy needs. Health education initiatives, user-friendly interfaces, clear instructions, and support resources can enhance the Digital Health Literacy of individuals and bridge the gap between technology and healthcare.

Unaffordable circumstances, poor socioeconomic status, lack of time to concentrate on self-managing diabetes, dietary confusion, and confusion about managing hypoglycemic as well as hyperglycemic conditions indicated the need to self-manage diabetes through a cost-effective means of Digital Health Solutions like a mHealth intervention that would alleviate the gaps in DSM.

Using a self-management app would boost its effectiveness and increase patients’ effectiveness in the self-management component in addition to the standard care provided by the doctor [48].

The T2DM patients’ usage of the mHealth app may help them regulate their calorie intake through a controlled diet and the presentation of low-calorie meals. People on insulin may also use the app to track their calorie consumption, which the doctor can use to establish the appropriate insulin dosage.

The use of several mobile health apps by medical professionals allowed them to gather clinical insights from diabetes patients and thus, revealed that mHealth apps could help people manage diabetes on their own [49].

## Tackling diabetes self-management by incorporating digital health solutions

The mHealth solutions offer a promising avenue for empowering individuals with diabetes to effectively manage their condition while helping the HCPs as well, to understand better treatment options for their patient’s well-being [50]. These apps allow for enhanced self-monitoring of Blood glucose and tracking, allowing for automatic data recording and visualization of trends over time. Users can also log food intake, physical activity levels, and medications, providing valuable insights into identifying patterns and tailoring self-management strategies. The tracked data allows sharing of the patient’s data with their physician on their follow-up hospital visit.

Interactive educational modules in mHealth apps deliver researcher-tailored educational content on healthy eating, exercise, and medication adherence, empowering users with knowledge for better self-care. Features with reminders and alerts for users to set personalized reminders for medication intake, blood glucose checks, and doctor appointments, promoting improved adherence to treatment plans. Telehealth consultations are another important aspect of mHealth platforms, they facilitate virtual consultations with healthcare providers, allowing for remote monitoring, feedback, and adjustments to treatment plans. Data sharing between patients and healthcare professionals enables better-informed decision-making and personalized care strategies. Improving health by engaging in better interactive gaming elements with rewards like badges for continuous use of the mHealth app and self-monitoring and tracking progress over time is helpful. Building social support communities via the mHealth platform, to connect users with other individuals living with diabetes, fostering peer support, knowledge sharing, and a sense of community involvement improves wellbeing.

The study’s limitations were that the other chronic conditions could not be assessed as the study focused on T2DM. The experience of the patient already using an app to manage his/her diabetes could not be assessed, in this study, as there were no participants who already used the app, the user experience would be helpful in understanding the technical limitations in the use of the app in making it more user friendly. Another limitation is that the need for a mHealth app for better self-management of T2DM is a perspective derived solely from the patients and the HCPs interviewed and thus reflects only the participants’ perspectives. Lastly, The data saturation was obtained at an early stage of the interviews, because of which the sample size was limited to 10 patients and 6 HCPs.

## Conclusion

The factors identified from this study are key to providing ultimate solutions to the addressed needs of the patient in improving their health and quality of life. MHealth solutions hold immense potential to revolutionize diabetes management by empowering individuals, facilitating communication with healthcare professionals, and promoting self-directed care. It’s important to note that further research is always valuable to solidify these findings and explore the specific features that contribute to these positive perceptions.

Digital health solutions can improve data accuracy, patient engagement, and treatment outcomes. However, successful implementation requires addressing technological barriers, and ensuring customizations of app features based on patient needs. Collaboration between technology developers, healthcare providers, and patients will be key to realizing the full benefits of digital health in diabetes management.

From the interviews conducted, the researchers were able to identify the needs and challenges faced by T2DM patients and get insight into the HCP’s perspectives on using a mHealth app among the patients to effectively manage T2DM in the future.

### Electronic supplementary material

Below is the link to the electronic supplementary material.

Supplementary Material 1

## Data Availability

No datasets were generated or analysed during the current study.
