# Supplementary material for: Identification of challenges and leveraging mHealth technology, with need-based solutions to empower self-management in type 2 diabetes: a qualitative study
Source: Diabetol Metab Syndr. 2024 Jul 30;16:182. doi: 10.1186/s13098-024-01414-9 (PMC11288030; doi:10.1186/s13098-024-01414-9)
Supplement: Supplementary file 1 — Supplementary Material 1 [file 13098_2024_1414_MOESM1_ESM.docx]

**Table 1- Patient Interview Guide**

| **Sl. No.** | **Interview Guide to T2DM Patients** |
| --- | --- |
|  | How did you know about your condition? Was it an accidental or intentional diagnosis? What practitioner did you meet? |
|  | How do you get in touch with your doctor for diabetes treatment?  Where did you access the information about the practitioner as well as on Diabetes? Name sites if any? From whom have you heard? |
|  | How do you manage diabetes in your day-to-day life? |
|  | Do you use any diabetic apps/ has anyone in your house using a diabetic app? If yes for how long? |
|  | What is your opinion on using a mHealth app for managing Diabetes? |
|  | What are your concerns about using a digital platform for feeding your health details? |
|  | Do you face any challenges in managing your diabetes? if yes, what are those? |
|  | What kind and how did you receive information about your disease and its management? Was that helpful? Or do you feel some information was missing? Which ones? |
|  | Do you feel confident, or do you experience stress when managing your diabetes? |
|  | Do you record the daily measures you’re doing? Is it easy? |

**Table 2-HCPs Interview Guide**

| **Sl. No.** | **Interview Guide to the HCPs on Type 2 Diabetes Management among Patients** |
| --- | --- |
|  | Could you describe the patient’s journey from their 1^st^ visit/diagnostic (frequency of visits, other professionals intervening) |
|  | What are the most frequently asked questions? Most frequent concerns? |
|  | Which resources do they use more often? What kind of help do they find around them? |
|  | What percentage of patients do you think are unable to access information/ get treated during their hospital visit? |
|  | What are your views about patients using the mHealth app for diabetes management? |
|  | What are your views on using a diabetic self-management app? |
|  | What do you think are the advantages and disadvantages of using an m app for the management of type 2 diabetes? |
